# Ultrasound-Guided Clavipectoral Fascial Plane Block Combined With Intermediate Cervical Plexus Block for Surgery on a Displaced Midshaft Clavicular Fracture in an Adolescent Athlete: A Case Report

**DOI:** 10.7759/cureus.64504

**Published:** 2024-07-14

**Authors:** Tatsuya Tsuji, Shun Takeuchi, Rei Tsuji, Hiroshi Nakano

**Affiliations:** 1 Department of Anesthesiology, Okazaki City Hospital, Okazaki, JPN; 2 Department of Anesthesiology and Intensive Care Medicine, Nagoya City University Graduate School of Medical Sciences, Nagoya, JPN

**Keywords:** case report, adolescent, clavicular fracture, multimodal analgesia, intermediate cervical plexus block, clavipectoral fascial plane block, ultrasound-guided, regional anesthesia

## Abstract

Ultrasound-guided clavipectoral fascial plane block (CPB) and intermediate cervical plexus block (ICPB) have been used as novel approaches for clavicular fracture surgery in adults. However, there are few reports of ultrasound-guided CPB combined with ICPB for clavicular surgery in children under 18 years of age. A 16-year-old male baseball player (weight, 57 kg; height, 160 cm) was scheduled to undergo open reduction and internal fixation with superior plate placement for a left-sided displaced midshaft clavicular fracture. We performed ultrasound-guided CPB using 0.25% ropivacaine (10 mL each) on the medial and lateral sides of the clavicle fracture between the periosteum of the clavicle and the clavipectoral fascia and ICPB using 0.25% ropivacaine (5 mL) under general anesthesia. The surgery proceeded smoothly, and the postoperative pain was minimal. In this case, ultrasound-guided CPB combined with ICPB was used effectively and safely to treat clavicular fractures in an adolescent athlete.

## Introduction

Ultrasound-guided clavipectoral fascial plane block (CPB) is a relatively novel regional anesthesia technique introduced by Valdes in 2017 [1]. In recent years, ultrasound-guided CPB and intermediate cervical plexus block (ICPB) have been used for regional analgesia and pain management of midshaft clavicle fractures in adults [2]. In adult patients undergoing clavicle surgery, CPB and ICPB are less likely to cause phrenic nerve paralysis and provide analgesic effects equivalent to those of ICPB and interscalene brachial plexus block (ISBPB) [2,3].

Clavicle fractures are common in both adults and adolescents [4], with 81% occurring in the midshaft region. Compared with adults, adolescent athletes have higher pain levels, greater anxiety, and more catastrophic thinking [5]. Therefore, post-surgical pain should be managed appropriately and effectively to avoid the transition to chronic pain and causing additional psychological burdens on young athletes.

However, few reports of ultrasound-guided CPB in pediatric patients exist. Here, we report safe and effective ultrasound-guided CPB combined with ICPB as a surgical block for midshaft clavicle fractures in an adolescent patient. We obtained written informed consent from the patient and his mother for conducting a novel pediatric regional anesthesia technique and publishing this case report.

## Case presentation

A 16-year-old male athlete (weight, 57 kg; height, 160 cm) was injured while playing baseball and diagnosed with a left displaced midshaft clavicle fracture. Because he requested to return to playing baseball as soon as possible, he was referred to our hospital four days after the injury for surgical intervention rather than conservative treatment. He had no other injuries except to the clavicle. His medical history and laboratory results were unremarkable (American Society of Anesthesiologists (ASA) physical status I). Open reduction and internal fixation with superior plate placement for a clavicular fracture were scheduled to be performed two weeks after the injury. We administered ultrasound-guided CPB combined with ICPB after the induction of general anesthesia to minimize physiological stress and pain during the perioperative period.

After establishing standard monitoring, including bispectral index (BIS) and neuromuscular monitoring, general anesthesia was induced with intravenous fentanyl (100 μg), propofol (target-controlled infusion of 3.0 μg/mL), and rocuronium (40 mg). The patient was intubated using an endotracheal tube (internal diameter 6.5 mm). 

**Figure 1:**
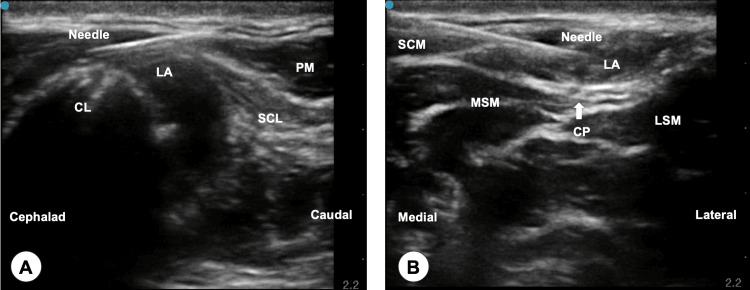
Ultrasound images of the clavipectoral fascial plane block (A) and the intermediate cervical plexus block (B) (A) Local anesthetic (10 mL of 0.25% ropivacaine) was deposited through the needle into the space between the periosteum of the clavicle and the clavipectoral fascia from caudal to cephalad using the in-plane technique; (B) A local anesthetic (5 mL of 0.25% ropivacaine) was deposited through the needle at the interfascial space between the sternocleidomastoid muscle and the prevertebral fascia in the medial-to-lateral direction using the in-plane technique. CL: clavicle; CP: cervical plexus; LA: local anesthetic; LSM: levator scapulae muscle; MSM: middle scalene muscle; PM: pectoralis major muscle; SCL: subclavian muscle; SCM: sternocleidomastoid muscle

Under sterile and aseptic conditions, ultrasound imaging was performed using a high-frequency linear array probe (6-13 MHz) (SonoSite SⅡ; Fujifilm SonoSite, Inc., Bothell, Washington, United States) after induction. Ultrasound-guided CPB combined with ICPB was performed to ensure adequate sensory coverage of the surgical field. A local anesthetic solution of 0.25% ropivacaine was used for both CPB and ICPB. To perform CPB, an ultrasound probe was applied to the medial and lateral aspects of the fracture site on the left anterior clavicle with the patient in the supine position. The in-plane technique was used to visualize a 22-gauge 5 cm ultrasound-visible needle (Ultraplex 360; B-Braun, Melsungen, Germany) inserted and advanced from the caudal to the cephalad direction. The position of the needle tip was confirmed by visible fluid spread into the space between the periosteum of the clavicle and the clavipectoral fascia, with 10 mL of local anesthetic solution injected at both the medial and lateral sites (Figure 1A). During ICPB, a linear probe was positioned on the left lateral side of the neck at the level of the cricoid cartilage, over the midpoint of the sternocleidomastoid muscle. The in-plane technique was applied to introduce the needle from medial to lateral, depositing 5 mL of 0.25% ropivacaine into the interfascial space between the sternocleidomastoid muscle and the prevertebral fascia (Figure 1B).

**Figure 2:**
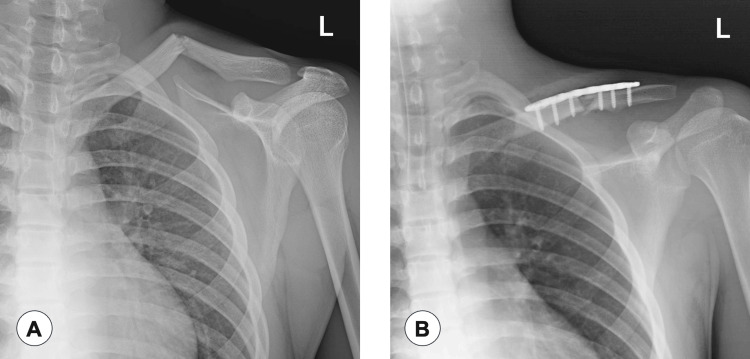
(A) Preoperative radiograph of the clavicle taken three days after the injury shows left midshaft clavicle fractures; (B) Postoperative radiograph of the clavicle taken immediately after open surgery to repair the left clavicle fractures.

After the CPB and ICPB procedures, the patient was placed in the beach chair position for surgery. General anesthesia was maintained with propofol (target-controlled infusion of 2.5-3.0 μg/mL) to maintain BIS between 40 and 60, and remifentanil (0.05-0.1 μg/kg/min) without circulatory change throughout most of the surgical procedure. Only when the surgeon drilled a hole in the clavicle to fix the plate did the heart rate temporarily increase from 69 to 80 beats/minute. Additional fentanyl (100 μg) was administered because the patient experienced pain at that time. Ondansetron (4 mg), dexamethasone (6.6 mg), flurbiprofen axetil (50 mg), and acetaminophen (1000 mg) were administered before surgery. The surgery was completed uneventfully, proceeded without complications, and lasted 77 minutes, with a total blood loss of 10 mL. After adequate spontaneous breathing, the patient emerged from anesthesia and was uneventfully extubated in the operating room. Figure 2 shows preoperative and postoperative radiographs of the left clavicle. 

After recovery from general anesthesia, the patient was transferred to the general ward of the Department of Orthopedic Surgery. Pain intensity was evaluated using a numerical rating scale (an 11-point scale, 0 = no pain; 10 = worst pain imaginable) before surgery and at one, six, 12, and 24 hours postoperatively, yielding scores of 2, 1, 1, 1, and 1, respectively. The patient received 400 mg of oral acetaminophen 12 hours after surgery and did not report any postoperative nausea or vomiting. The postoperative course was uneventful, with no respiratory complications, and the patient was satisfied with the postoperative analgesia. The patient was discharged on the second postoperative day without any adverse events.

## Discussion

This case report demonstrates the use of ultrasound-guided CPB combined with ICPB for midshaft clavicular fractures in an adolescent athlete. As skin sensory blockade could not be achieved with CPB alone [2], we also performed ICPB to block the supraclavicular branch of the superficial cervical plexus. The advantages of regional anesthesia in pediatric patients (from preterm to teenagers) include reduced opioid consumption, incidence of postoperative nausea and vomiting, postoperative pain scores, and incidence of respiratory complications [6]. In this case, there were no postoperative anesthesia-related complications, and postoperative pain was effectively controlled with minimal intraoperative opioid administration. CPB and ICPB are technically straightforward and provide excellent analgesia during clavicle surgery, which is typically uncomfortable and painful for pediatric patients. Although regional anesthesia in pediatric patients has increased significantly in the past decade, many studies of new regional anesthesia techniques have been conducted in adults rather than children [7]. Therefore, it is essential to continually assess the safety and efficacy of newly implemented regional anesthesia techniques in adults in order to inform their use in pediatric patients. 

A previous study reported that teenage patients (11-18 years) admitted to surgical units had an incidence of moderate to severe pain higher than that in infants and young children (1-10 years) (38%, 32%, and 17%, respectively, for teenagers, infants, and young children) [8]. Additionally, the report indicated that the low use of regional anesthesia might have led to higher pain scores, and the majority of children with moderate or severe pain might experience persistent clinically significant pain in subsequent days. Therefore, providing appropriate regional anesthesia for surgery to high-risk adolescent populations is critical. In this case, we selected CPB combined with ICPB under general anesthesia as part of a multimodal analgesic regimen for several reasons. 

First, we needed to provide favorable postoperative analgesia with a low risk of paralysis because the athlete patient was urgently requested to return to baseball as soon as possible. ISBPB combined with ICPB has traditionally been used in clavicular fracture surgery [9]. Although this combination of ISBPB and ICPB can effectively meet surgical needs, ISBPB can cause complications such as the diaphragm and upper arm paralysis [2,3]. In recent years, the combination of CPB and ICPB for clavicle surgery has been reported to offer longer postoperative analgesia and better preservation of upper-limb motor function than ISBPB and ICPB [3]. Furthermore, severe and persistent nerve injuries in a teenage athlete (football goalkeeper) caused by ultrasound-guided continuous ISBPB have been reported [10]. To our knowledge, no cases of severe and persistent neurological disability have been reported after CPB or ICPB in children. Therefore, we selected CPB and ICPB instead of ISBPB and ICPB as part of the multimodal analgesic regimen. 

Second, we performed general anesthesia with CPB and ICPB. Patients undergoing awake regional anesthesia have been reported to experience cardiovascular instability, including bradycardia and hypotension [11]. Although anesthesiologists typically avoid general anesthesia with positive pressure ventilation in cases of coexisting pneumothorax due to trauma, this patient had no pneumothorax. Therefore, general anesthesia was used to prevent adverse reflexes with sympathetic blockade or overstimulation of the sympathetic nervous system caused by awake regional anesthesia in the beach chair position.

Third, the analgesic effects of CPB as a novel regional anesthesia technique remain controversial. The clavicle and clavicular joints are innervated by the subclavian, lateral pectoral, and supraclavicular nerves [12]. CPB has been thought to relieve pain by blocking branches of the sensory nerve in the clavicular fascia and plane [2]. However, recent cadaver studies have indicated that the injected solution does not achieve complete dispersion within the periclavicular region, regardless of the CPB approach [13,14]. These studies revealed that methylene blue distributed predominantly to the anterosuperior area, with minimal impact on the posteroinferior region at the clavicular plane [14]. This suggests that the pain associated with the posterior clavicle drilling may have been inadequately controlled in this case as well. Consequently, performing clavicular surgery with CPB and ICPB alone without general anesthesia may be challenging in adolescents.

Adolescents have higher pain scores and greater anxiety compared with adults [5], necessitating appropriate regional anesthesia under general anesthesia for clavicle surgery. As the adolescent in the current case was of a similar size as an adult, the CPB and ICPB techniques themselves did not present any particular difficulties and could be performed safely. Moreover, we consider that CPB and ICPB were effective for pain control in multimodal analgesia, as the need for analgesics (400 mg of oral acetaminophen) arose 12 hours after surgery when the analgesic effect of single-shot CPB and ICPB appeared to diminish.

## Conclusions

Administration of appropriate regional anesthesia to adolescents at high risk for pain before surgery is critical. However, ultrasound-guided CPB and ICPB in pediatric patients have rarely been reported. CPB and ICPB provide highly efficacious analgesia during clavicle surgery, which is typically uncomfortable and painful for pediatric patients without reports of complications such as severe nerve injuries. Moreover, CPB is less likely to cause phrenic nerve paralysis in patients undergoing clavicle surgery. In this case, ultrasound-guided CPB combined with ICPB was effectively and safely used as part of a multimodal analgesic regimen for left midshaft clavicle fractures in an adolescent athlete. Further studies are warranted to assess the efficacy and safety of CPB combined with ICPB in younger patients.
